# Society Meetings

**Published:** 1896-09

**Authors:** 


### Society Meetings.

The American Academy of Railway Surgeons will hold its third annual meeting at Chicago, in the Auditorium hotel, Wednesday, Thursday and Friday, September 23, 24 and 25, 1896, under the presidency of Dr. John E. Owens, of Chicago. A detailed program has been issued, that indicates preparations for an elaborate meeting. Dr. E. M. Dooley, of Buffalo, is announced to read a paper on Fracture of the femur, and Dr. C. M. Daniels, of Buffalo, is a member of the executive board.

PROVISIONAL PROGRAM.

First session will open on Wednesday, September 23d, at 10 A. M., standard time.

President's address, J. E. Owens, M. D., Chicago, Ill.

*Executive Session.*—1. Use and abuse of expert testimony, with some recommendations for its improvement, by an attorney. 2. Diseases of railway men caused by their occupation, J. F. Pritchard, M. D., district surgeon, C. & N. W. R'y, Manitowoc, Wis.; discussion opened by J. H. Ford, M. D., chief surgeon, "Big Four" R'y, Wabash, Ind. 3. The personal equation among train men; its importance equal or greater than the color sense; illustrated by an automatic machine which records both, Robert Tilley, M. D., Chicago; discussion opened by F. B. Eaton, M. D., oculist, O. R. & N. Co., Portland, Oregon.

*Second Session.*—Wednesday, September 23d, at 2.30 P. M., standard.

1. Penetrating wounds of the eye-ball, Archibald G. Thompson, M. D., assistant oculist, Penna. R'y, Philadelphia, Pa. 2. Penetrating wounds of the eye-ball, G. A. Wall, M. D., eye and ear surgeon, A. T. & S. Fe R'y, Topeka, Kan. 3. Penetrating wounds of the eye-ball, T. J. Redelings, M. D., surgeon, C. & N.

W. R'y, Marinette, Wis. 4. Penetrating wounds of the eye-ball, with special reference to differential diagnosis, D. C. Bryant, M. D., oculist, U. P. R'y, Omaha, Neb.; discussion opened by August Rhu, M. D., surgeon, "Big Four" R'y, Marion, Ohio. 5. Remote effects of bone trauma, D. S. Fairchild, M. D., surgeon, C. &. W. R'y, Clinton, Iowa; discussion opened by K. A. J. McKinzie, chief surgeon, O. R. & N. Co., Portland, Oregon.

*Third Session.*—THURSDAY, SEPTEMBER 24th, at 9.30 A. M., standard.

*Executive Session.*—Report of secretary and treasurer. Report of standing committees. Registration of fellows and payment of dues. Consideration and disposal of applications for fellowship. Miscellaneous business.

1. Medico-legal aspects of floating kidney, R. Harvey Reed, M. D., consulting surgeon, B. & O. R'y, Columbus, Ohio; discussion opened by W. J. Galbraith, M. D., chief surgeon, U. P. R'y, Omaha, Neb. 2. Railway surgery, Jessie Hawes, M. D., division surgeon, U. P. D. & G. R'y, Greeley, Colo.; discussion opened by R. L. Harris, chief surgeon, G. & St. A. R'y, Oakland, Fla. 3. Emergency surgical practice, C. K. Cole, M. D., chief surgeon, Montana Central R'y, Helena, Mont.; discussion opened by Truman Miller, M. D., chief surgeon, C. & G. T. R'y, Chicago, Ill.

*Fourth Session.*—THURSDAY, SEPTEMBER 24th, at 2.30 P. M., standard.

1. First aid in railway emergencies, James E. Pilcher, M. D., U. S. barracks, Columbus, Ohio; discussion opened by G. W. Hogeboom, M. D., chief surgeon, A. T. & S. Fe R'y, Topeka, Kan. 2. Experimental research into shock in abdominal operations and injuries, Geo. W. Crile, M. D., surgeon, C., C., C. & St. L. R'y, Cleveland, Ohio; discussion opened by J. W. Perkins, M. D., surgeon, U. P. R'y, Kansas City, Mo. 3. Shock and collapse, with special reference to amputations, Webb J. Kelly, M. D., surgeon, "Big Four" R'y, Galion, Ohio; discussion opened by W. L. Estes, M. D., chief surgeon, Lehigh Valley R'y, South Bethlehem, Pa. 4. The delirium of shock, R. S. Harnden, M. D., surgeon, Erie R'y, Waverly, N. Y.; discussion opened by Milton Jay, M. D., chief surgeon, C. & E. I. R'y, Chicago, Ill. 5. Injuries of the hands and fingers, John McLean, M. D., chief surgeon P. P. C. Co., Pullman, Ill.; discussion opened by W. L. Buechner, M. D., surgeon N. Y., L. E. & W. R'y, Youngstown, Ohio.

*Fifth Session.*—FRIDAY, SEPTEMBER 25th, at 9.30 A. M., standard.

1. An experimental study of Colles' and Potts' fractures on the cadaver, A. D. Bevan, M. D., chief surgeon, Iowa Central R'y, Chicago; discussion opened by R. Ortega, M. D., chief surgeon, Mexican Int. R'y, C. P., Diaz, Mex. 2. The cause and mechanical treatment of subluxation of the knee-joint, S. L. McCurdy, M. D., surgeon, Penna. Central, Pittsburg, Pa.; discussion opened by L. E. Lemen, M. D., division surgeon, U. P. R'y, Denver, Colo. 3. Compound comminuted fractures at the knee, with report of a case, W. A. Ward, M. D., surgeon, N. Y. C. & St. L. R'y, Conneaut, Ohio; discussion opened by A. C. Scott, M. D., chief surgeon, G. C. & S. F. R'y, Temple, Texas. 4. Relation of tuberculosis of the knee to injuries of said joint, H. Reineking, M. D., surgeon, C. & N. W. R'y, Sheboygan, Wis.; discussion opened by W. H. Buechner, M. D., surgeon, Erie R'y, Cleveland, Ohio.

*Sixth Session.*—FRIDAY, SEPTEMBER 25th, at 2.30 P. M., standard.

*Executive Session.*—Election of officers. Selection of next place of meeting. Miscellaneous business.

1. Fractures of the femur, E. M. Dooley, M. D., surgeon, N. Y., L. E. & W. R'y, Buffalo, N. Y.; discussion opened by J. I. Darby, M. D., chief surgeon, A. & M. R'y, Americus, Ga. 2. Past and present obstacles to the radical cure of hernia, E. Wyllys Andrews, M. D., surgeon, Wabash R'y, Chicago; discussion opened by R. D. Mussey, M. D., chief surgeon, C. H. & D. R'y, Cincinnati, Ohio. 3. Treatment of tramps and trespassers, H. J. Williams, M. D., surgeon, Central R'y, Macon, Ga.; discussion opened by Fred J. Hodges, M. D., chief surgeon, C. & S. E. R'y, Anderson, Ind. 4. Röntgen ray demonstration. Introduction of officers-elect. Adjournment.

Arrangements are being completed by the chairman, Dr. W. J. Galbraith, Omaha, Neb., for the transportation of fellows to the next annual meeting. Hotel accommodations will be arranged for by Dr. A. D. Bevan, Chicago, Ill., chairman committee of arrangements.

Other papers will be added to the program when the final announcement is made. Those interested in the advancement of railway surgery are respectfully invited to attend and participate in the discussions.

The American Dermatological Association will hold its twentieth annual meeting at the Hot Sulphur Springs of Virginia, September 8, 9 and 10, 1896, under the presidency of Dr. A. R. Robinson, of New York. The following program has been prepared by the secretary, Dr. C. W. Allen, of New York :

FIRST DAY—TUESDAY, SEPTEMBER 8, 1896.

Business meeting (with closed doors) at 9.30 A. M.: Report of council ; nomination of officers for the ensuing year ; appointment of auditing committee ; proposals for active and honorary membership ; miscellaneous business.

Scientific session at 10.30 A. M.: 1. Address by the president, A. R. Robinson ; 2. Notes on dermatitis venenata, Dr. J. G. White ; 3. Paget's disease of the nipple, Dr. G. T. Jackson ; 4. Ulcerative scleroderma, Dr. P. A. Morrow ; 5. Two cases of arsenical pigmentation, Dr. W. A. Hardaway.

Scientific session at 3 P. M.: 6. A pathological and clinical classification of the diseases of the skin, Dr. L. A. Duhring ; 7. Erythema multiforme, with a report of two cases, Dr. W. T. Corlett ; 8. A peculiar affection of the mucous membrane of the lips and mouth, with colored drawings and photographs, Dr. J. A. Fordyce ; 9. A favus-like eruption of the oral mucous membrane, caused by the aspegillus niger, Dr. J. MacF. Winfield.

SECOND DAY—WEDNESDAY, SEPTEMBER 9, 1896.

Business meeting, with closed doors, at 9.30 A. M.: Report of treasurer and auditing committee ; election of officers ; election of active and honorary members ; selection of time and place of next meeting ; miscellaneous business.

Scientific session at 10.30 A. M.: General discussion—10. What effect do diet and alcohol have upon the causation and course of the eczematous affections and psoriasis ? Discussion opened by Dr. J. C. White ; 11. Cases of mycosis, fungoides and sarcomatosis, Dr. J. T. Brown ; 12. Xanthoma Diabeticorum, Dr. A. R. Robinson ; 13. Some glycosuric dermatoses, Dr. C. W. Allen ; 14. A case of hypertrophic rosacea (pachydermatosis) resembling tubercular leprosy, cured with thyroid extract.

Scientific session at 7.30 P. M.: 15. Exhibition of drawings, water colors, colored photographs, photographs and portraits of rare cases ; 16. Presentation of instruments, specimens and demonstrations of microscopic preparations and photomicrographs.

THIRD DAY—THURSDAY, SEPTEMBER 10, 1896.

Morning session at 9.30 A. M.: 17. The relation of dermatitis hepetiformis to certain other diseases, Dr. L. A. Duhring; 18. Bath pruritus, Dr. H. W. Stelwagon; 19. Eruption from the local use of iodoform (with colored drawings), Dr. J. A. Fordyce; 20. Impetigo contagiosa universalis, Dr. C. W. Allen. Adjournment.

---

THE ninth annual meeting of the American Association of Obstetricians and Gynecologists, as announced in the JOURNAL for August, will be held at the Hotel Jefferson, Richmond, Va., Tuesday, Wednesday and Thursday, September 22, 23 and 24, 1896.

The following additional papers have been presented, the numbers being consecutive to the first list: 35. Three cases of bowel resection, end to end suture, recovery, John B. Deaver, Philadelphia; 36. Tubercular peritonitis, W. A. Jayne, Denver; 37. Title to be announced, T. J. Crofford, Memphis; 38. Surgical management of acute diffuse peritonitis, with report of cases, John Young Brown, Jr., St. Louis; 39. Ventro-fixation and Alexander's operation, Geo. Ben Johnston, Richmond; 40. Rupture of the uterus during labor, with a specimen, B. W. Hypes, St. Louis; 41. Ten abdominal hysterectomies for fibroid disease of the uterus, Reuben Peterson, Grand Rapids; 42. Porro's operation, with report of a case, Edwin Ricketts, Cincinnati; 43. Methods of dealing with the stump in operations for appendicitis, C. N. Smith, Toledo; 44. Report of nine cases of uterine fibroids complicated by pregnancy, M. Rosenwasser, Cleveland; 45. Abdominal section for tubercular disease, Thos. E. McArdle, Washington; 46. Atresia with retention of the menses, treatment, W. H. Myers, Fort Wayne; 47. Suture of large vessels injured in operation, with demonstration of method, J. B. Murphy, Chicago; 48. Plastic surgery of the male genital tract, Joseph Price, Philadelphia; 49. The Limitations of nephrorrhaphy, Hugh M. Taylor, Richmond; 50. Intestino-vaginal fistulæ, with report of a case, C. C. Frederick, Buffalo; 51. Shall we leave the uterus *in situ* after the excision of the adnexa? E. F. Fish, Milwaukee; 52. Memorial of Dr. Hiram Corson, Traill Green, Easton.

---

THE Mississippi Valley Medical Association will hold its next annual meeting at St. Paul, September 15, 16, 17 and 18, 1896, under the presidency of Dr. H. O. Walker, of Detroit. The date of this meeting has been changed from October, in order to permit members and their families to avail themselves of the opportunity to make a tour through the Yellow Stone Park. An excursion is expected to leave St. Paul, Friday evening, September 18th, arriving at Mammoth Hot Springs the following Sunday. The excursionists will be enabled to devote five days to inspecting the wonders of the Yellow Stone, reaching St. Paul, on the return trip, September 27th. Details will be duly announced.

In this connection we are permitted to state that the Chicago, Milwaukee & St. Paul railway will be the official route of the association from Chicago. A reduction of rates is expected that will include points as far east as Buffalo. Mr. William Kelly, in the Ellicott Square building, represents the aforementioned railway in this city, and will gladly furnish any information concerning the proposed meeting and the subsequent excursion.

---

THE American Public Health Association will hold its twenty-fourth annual meeting at Buffalo, in the Ellicott Square building, Tuesday, Wednesday, Thursday and Friday, September 15, 16, 17 and 18, 1896, under the presidency of Dr. Eduardo Licéaga, of the City of Mexico.

All persons interested in the preservation of public health, whether doctors, lawyers, ministers, civil and sanitary engineers, or business men, are cordially invited to attend the sessions. Dr. Ernest Wende is chairman of the local committee of arrangements, and will open an office in the Ellicott Square some days before the meeting, for the purpose of giving information on the subject.

---

THE Tri-State Medical Society, of Alabama, Georgia and Tennessee, will hold its eighth annual meeting at Chattanooga, October 13, 14 and 15, 1896, under the presidency of Dr. J. B. Murfree, of Murfreesboro'. The secretary, Dr. Frank Trester Smith, of Chattanooga, is preparing an elaborate program.

---

THE American Electro-Therapeutic Association will hold its sixth annual meeting, Tuesday and Wednesday, September 29 and 30, and Thursday, October 1, 1896, in the Austin hall, Studio building, Clarendon street, near St. James avenue, Boston, Mass.

The American Association for the Advancement of Science is holding an interesting and well-attended meeting while these pages are printing. An elaborate program has been prepared and the several sections are in full working order. An address of welcome on behalf of the Buffalo Society of Natural Sciences was delivered by Dr. Roswell Park, and many scientific papers of great value are being read and discussed. The distinguished paleontologist, Prof. Edward D. Cope, of Philadelphia, is the president.

Among other things Dr. Park, in his address of welcome, said:

I appreciate the honor of extending to you a welcome to our fair city, pleasant in climate, bustling with commercial activity and the pride of more than one-third of a million of inhabitants. Not the least of our pleasant recollections in seeing you now is the remembrance that after the period of your inactivity during the civil war, your first meeting for reorganisation was held at Buffalo. Twice since then your association has honored us by selecting Buffalo as a meeting place, and now we again extend to you a welcome bounded only by the city's limits and the hospitality of its citizens. We are proud of our city, of its commercial prosperity, of its educational institutions and natural beauties. We would ask those who have been here before to compare our present with our past, and see with what rapid strides we are becoming one of the largest and most prosperous cities of the continent.

In responding, President Cope said that each member of the association felt the warm sympathy and the hearty hospitality that had been extended by the people of Buffalo. He spoke of original research as promoting mainly the advancement of science. Love of research, he said, makes a man devote himself to the study of science, and it means endless advantages both to the investigator and to those who profit by his research. The scientific career is one which offers great advantages in this country, rich in the elements that make the calling a source of delight. It is a life of happiness, for in congenial labor is found happiness. In the line of intellectual progress the scientific career offers the very best.

